# Ovariectomy and chronic stress lead toward leptin resistance in the satiety centers and insulin resistance in the hippocampus of Sprague-Dawley rats

**DOI:** 10.3325/cmj.2016.57.194

**Published:** 2016-04

**Authors:** Vedrana Ivić, Senka Blažetić, Irena Labak, Marta Balog, Luka Vondrak, Robert Blažeković, Sandor G. Vari, Marija Heffer

**Affiliations:** 1 Josip Juraj Strossmayer University of Osijek, Faculty of Medicine Osijek, Department of Medical Biology and Genetics, Osijek, Croatia; 2 Josip Juraj Strossmayer University of Osijek, Department of Biology, Osijek, Croatia; 3 Department of Cardiac and Transplantation Surgery, University Hospital Dubrava, Zagreb, Croatia; 4 Josip Juraj Strossmayer University of Osijek, Faculty of Medicine Osijek, Department of Surgery and Neurosurgery, Osijek, Croatia; 5 International Research and Innovation in Medicine Program, Cedars–Sinai Medical Center, Los Angeles, CA, USA

## Abstract

**Aim:**

To evaluate the changes in the expression level of gonadal steroid, insulin, and leptin receptors in the brain of adult Sprague-Dawley female rats due to ovariectomy and/or chronic stress.

**Methods:**

Sixteen-week-old ovariectomized and non-ovariectomized female Sprague-Dawley rats were divided in two groups and exposed to three 10-day-sessions of sham or chronic stress. After the last stress-session the brains were collected and free-floating immunohistochemical staining was performed using androgen (AR), progesterone (PR), estrogen-β (ER-β), insulin (IR-α), and leptin receptor (ObR) antibodies. The level of receptors expression was analyzed in hypothalamic (HTH), cortical (CTX), dopaminergic (VTA/SNC), and hippocampal regions (HIPP).

**Results:**

Ovariectomy downregulated AR in the hypothalamic satiety centers and hippocampus. It prevented or attenuated the stress-specific upregulation of AR in these regions. The main difference in stress response between non-ovariectomized and ovariectomized females was in PR level. Ovariectomized ones had increased PR level in the HTH, VTA, and HIPP. Combination of stressors pushed the hypothalamic satiety centers toward the rise of ObR and susceptibility to leptin resistance. When exposed to combined stressors, the HIPP, SNC and piriform cortex upregulated the expression of IR-α and the possibility to develop insulin resistance.

**Conclusion:**

Ovariectomy exacerbates the effect of chronic stress by preventing gonadal receptor-specific stress response reflected in the upregulation of AR in the satiety and hippocampal regions, while stress after ovariectomy usually raises PR. The final outcome of inadequate stress response is reflected in the upregulation of ObR in the satiety centers and IR-α in the regions susceptible to early neurodegeneration. We discussed the possibility of stress induced metabolic changes under conditions of hormone deprivation.

All cells in the body are working together in order to maintain homeostasis or physiological variance of conditions that support a multitude of special functions. When the physiological variance is violated we say that the body is under stress. Any external or internal condition that disturbs the homeostasis is considered a stressor (1). The body can cope with stressors if they are short and act suddenly. Both short-term (acute) and long-term (chronic) stress affect the hypothalamus-pituitary-adrenal axis (HPA) and the sympathetic autonomic nervous system (SNS). The joined outcome of the two stress-related systems is the increase of adrenal glucocorticoids, which leads to enhanced metabolism and cognition, while immune and reproductive systems are inhibited (2,3). These changes are known as the stress response and they are beneficial in short periods of time. When stressors act chronically these changes may lead to reduction of the HPA and SNS stress resilience and a failure in the maintenance of homeostasis. Chronic stress is increasing the risk for metabolic disorder and cardiovascular disease (CVD), characterized by high blood pressure, obesity, and consequently hormonal imbalance (4). Stress response is sex specific and regulated by gonadal steroid hormones (5,6). In general, women manage stressors better, but only till the period of menopause, because of the presence of protective gonadal steroid hormones (7). Menopause is accompanied by weight gain and obesity, and menopausal women eventually exhibit the characteristics of CVD and metabolic syndrome (8). Obesity, in particular abdominal phenotype, occurs as a response to chronic stress (9). Obesity is a result of an imbalance between the mechanisms controlling energy intake and energy consumption. Overall, long-term energy balance at the level of organism and individual cell is controlled by hormones leptin and insulin (10,11). Leptin is a hormone produced by the fat tissue which acts via its receptor ObR (12,13). It was noted that leptin suppresses HPA axis (14) and has an impact on stress-related behavior (15). Insulin is a pancreatic hormone whose level positively correlates with the amount of fat (11,16). Ovariectomy promotes menopause in the animal models (17). High fat diet and ovariectomy increase ObR expression in the lateral hypothalamic nuclei and barrel cortex (18).

Contrary to the energy intake, which is governed by well described hypothalamic nuclei and peptide hormones that control them, energy consumption is related to the pattern of behavior governed by expression of gonadal steroids in the brain. The gonadal steroids influence gene expression in the central nervous system, and in this way they change reproductive behavior and behavior overall (19). Particularly responsive to gonadal steroids are neural circuits regulating autonomous functions, food seeking behavior, and memory (20-22). We have previously shown that chronic stress induces changes in the distribution of the receptors for gonadal steroids at the level of adrenal gland (23) and also proposed that certain changes induced by chronic stress also happen in the brain. The aim of this study was to evaluate changes in the expression level of receptors for gonadal steroids, ObR and insulin receptor alpha (IR-α), in the brain of adult Sprague-Dawley female rats due to ovariectomy and/or chronic stress.

## Material and methods

### Experimental animals

**Figure 1:**
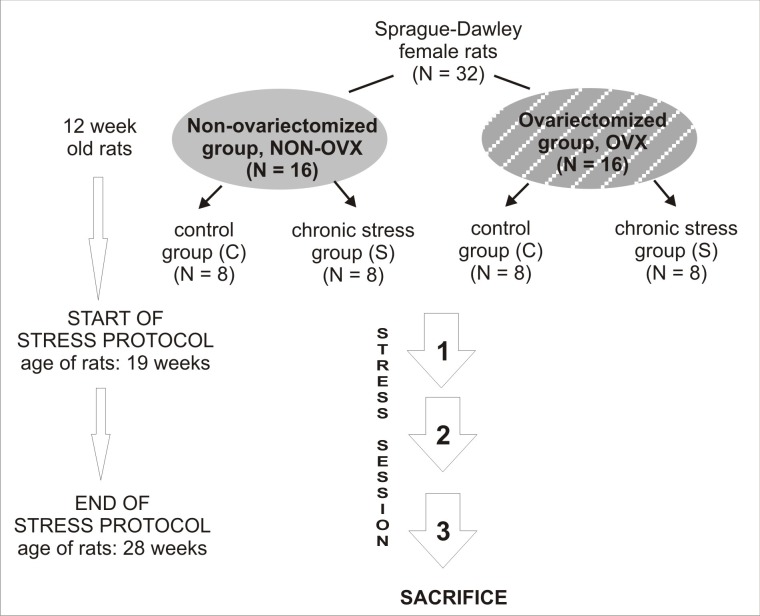
Experimental animals and chronic stress protocol.

This study was performed at the Animal Facility of the Faculty of Medicine Osijek and was approved by the Ethics Committee of the Croatian Ministry of Agriculture, approval number: 2158-61-07-11-51. Thirty two 16-week-old female Sprague-Dawley rats were divided in two groups: non-ovariectomized (NON-OVX) and ovariectomized (OVX). These groups were subdivided into chronic stress and control group (Figure 1). Each group consisted of 8 animals that were housed within standard laboratory setting. Standard laboratory rat food and tap water were available *ad libitum *except in cases when food deprivation was a stressor. All procedures were carried out in agreement with EU Directive on Laboratory Animals.

### Ovariectomy and chronic stress protocol

Ovariectomy was performed at the age of 12 weeks. All surgical procedures regarding ovariectomy were performed on the same day on all animals by a surgeon proficient in such procedures. The Harlan protocol was followed (Harlan HUS-QREC-PRD-932, Issue: 01, Revision 03). Animals were anesthetized with isoflurane (Forane® isofluranum, Abbott Laboratories Ltd, Queenborough, UK). After the procedure, food and tap water were provided *ad libitum*. Animals were intensively monitored 72 hours after the procedure. In previous studies we showed that if ovariectomy was performed 4 weeks before the stress protocol, the stress caused by surgical procedure can be considered as irrelevant and fully compensated. This strategy allowed us to omit sham operated animals and reduce the total number of animals used. There was no difference in the behavioral response of OVX animals. Together with chronic stress protocol, we performed acute stress protocol in one part of the animals. The samples from this part of the experiment were analyzed and published by our Hungarian collaborators who measured gonadal steroid hormones in the serum of ovariectomized animals and confirmed the procedures (24).

Animals were exposed to chronic stress in three sessions lasting 10 days each, according to Balog et al (23). In short, when rats turned 19 weeks, stress (S) animal groups were exposed to a combination of various stressors, such as cold restraint, food deprivation, irregular noise, and others stressors. Control (C) animal groups were exposed to the same environment and were handled equally, but the stressor was not present. After chronic stress protocol completion, animals were of 28 weeks age. Animal body weight was measured at the beginning of the study and after each session (Figure 1).

### Tissue sampling

Animals were sacrificed after the last chronic stress session. They were anesthetized with the combination of Ketamine IM (Ketanest, Pfizer Corporation, New York City, NY, USA; concentration: 30 mg/kg) and inhalation gas (Forane® isofluranum, Abbott Laboratories Ltd, Chicago, IL, USA). The brains were isolated, fixed with 4% paraformaldehyde for 24h, sucrose cryoprotected, frozen by immersion in precooled isopentane and stored at -80°C till analysis.

### Immunohistochemistry

Free-floating immunohistochemistry was performed on 35 μm-thick coronal brain sections. After pretreatment with 1% H_2_O_2_ for inactivation of endogenous peroxidase activity, sections were submitted to the blocking solution containing 1% bovine serum albumin and 5% goat serum in 1xPBS for two hours at +4°C. Afterwards sections were incubated with primary antibodies for 42 h at +4°C. Primary antibodies, all from the Santa Cruz Biotechnology, Dallas, TX, USA, were prepared in blocking solution and used in different dilutions: anti-androgen receptor (AR; 1:250) (#SC-816; N-20), anti-progesterone receptor (PR; 1:100) (#SC-538; C-19), anti-estrogen receptor beta (ER-β; 1:100) (#SC-8974; H-150), anti-leptin receptor (ObR; 1:50) (#SC-8325; H-300), and anti-insulin receptor alpha (IR-α) (#SC-710; N-20). The sections were rinsed with 1xPBS and incubated for 4 h at +4°C with secondary biotinylated goat anti-rabbit antibody (#115-065-144, Jackson Immuno Research, West Grove, PA, USA) diluted in blocking solution. After rinsing with 1xPBS, the sections were incubated with VECTASTAIN® Elite® ABC kit (#PK-6100 Vector Laboratories Inc., Burlingame, CA, USA) for 2 h at +4°C. Rinsing of sections with 1xPBS was followed by incubation with peroxidase substrate kit (Vector Laboratories Inc.). Immunohistochemistry was repeated at least 3 times for each antibody, each time on a different sample from the same group. After mounting on slides, the sections were air-dried and coverslipped with VectaMount Permanent Mounting Medium (#H-5000, Vector Laboratories Inc.). Under 200 × magnification, an area of 0.02mm^2^ of 5 brain regions was imaged with Olympus D70 camera (Olympus, Hamburg, Germany) set up on Zeiss Axioskop 2 MOT microscope (Carl Zeiss Microscopy, Thornwood, NY, USA).

### Counting of immunopositive cells

This study analyzed distribution of gonadal steroid receptors: AR, PR, ER-β, and receptors for hormones in charge of long-term energy balance maintenance: ObR and IR-α. The immunopositive cells (both neurons and glia) were counted using ImageJ software (US National Institutes of Health, Bethesda, MD, USA) in five rat brain regions: hypothalamus (HTH), cortex (CTX), hippocampus (HIPP), and two dopaminergic areas – ventral tegmental area (VTA) and *substantia nigra pars compacta* (SNC). Counting of each slide was performed by 3 different persons, using coded numbers to prevent bias.

### Statistical analysis

The distribution of data was determined by Shapiro-Wilk test. As the distribution was not normal, comparisons of specific sets of two subgroups were conducted using Mann-Whitney test. The data used for comparison are shown in Supplemental Tables. The following sub-groups were compared: (A) NON-OVX-C and OVX-C groups were compared to observe the effect of ovariectomy, (B) NON-OVX-C and NON-OVX-S groups were compared in order to notice changes due to chronic stress exposure, (C) OVX-C and OVX-S groups were compared to determine the effect of chronic stress in ovariectomized female rats, particularly to determine if the direction of changes was similar as in non-ovariectomized females, (D) NON-OVX-S and OVX-S groups were compared to observe the influence of ovariectomy on the response to chronic stress and (E) NON-OVX-C group was compared to OVX-S group in order to reveal combined impact of ovariectomy and chronic stress. Statistical tests were performed using the statistical software package SPSS (SPSS Inc. Released 2008. SPSS Statistics for Windows, Version 13.0, Chicago, IL, USA). Statistical significance level was set at *P* < 0.01.

## Results

The body weight of animals followed the same pattern described in the previous publication. Significant changes in body weight were not observed based only on ovariectomy or chronic stress. Significant increase in body weight was noticed just in combination of ovariectomy and chronic stress after the first (OVX-S vs NON-OVX-S, *P* = 0.021) and second (OVX-S vs OVX-C, *P* = 0.031), but not after the third stress session (23).

The comparisons of specific sets of two animal sub-groups revealed significant differences described further in the text. The P-values of corresponding significant changes are shown in the Tables 1, 2, and 3 (indicated in text, as in the tables, with letters A, B, C, D, and E). The data used to compare animal sub-groups are presented in the Supplement (Supplemental Tables 1-5).

**Table 1:** The evaluation of change in expression level of gonadal steroid receptors

			NON-OVX-C vs OVX-C, (A)*	NON-OVX-C vs NON OVX-S, (B)*	OVX-C vs OVX-S, (C)*	NON-OVX-S vs OVX-S, (D)*	NON-OVX-C vs OVX-S, (E)*
**HTH** **Satiety for homeostasis**	**ARC**	**AR**	**-**	**↑, *P* = 0.001**	**-**	**↓, *P* = 0.001**	**-**
		**PR**	**-**	**-**	**-**	**-**	**-**
		**ER-β**	**-**	**-**	**-**	**↓, *P* = 0.002**	**↓, *P* = 0.003**
	**LH**	**AR**	**↓, *P* = 0.001**	**-**	**-**	**-**	**↓, *P* = 0.001**
		**PR**	**-**	**-**	**↑, *P* = 0.001**	**↑, *P* = 0.001**	**↑, *P* = 0.001**
		**ER-β**	**-**	**-**	**-**	**-**	**-**
	**PV**	**AR**	**↓, *P* = 0.003**	**↑, *P* = 0.003**	**↑, *P* = 0.001**	**↑, *P* = 0.001**	**-**
		**PR**	**-**	**-**	**-**	**-**	**-**
		**ER-β**	**↑, *P* = 0.001**	**-**	**↓, *P* = 0.004**	**-**	**-**
**Feeding for reward**	**VTA**	**AR**	**↑, *P* = 0.001**	**-**	**↓, *P* = 0.002**	**-**	**↑, *P* = 0.001**
		**PR**	**↑, *P* = 0.006**		**↑, *P* = 0.002**	**↑, *P* = 0.007**	**↑, *P* = 0.001**
		**ER-β**	**↓, *P* = 0.001**	**-**	**↑, *P* = 0.001**	**-**	**-**
	**PIR**	**AR**	**↓, *P* = 0.001**	**-**	**↑, *P* = 0.007**	**-**	**↓, *P* = 0.002**
		**PR**	**-**	**-**	**-**	**-**	**-**
		**ER-β**	**-**	**-**	**-**	**-**	**-**
**Non-declarative memory**	**SNC**	**AR**	**-**	**-**	**-**	**-**	**-**
		**PR**	**-**	**↑, *P* = 0.002**	**-**	**-**	**-**
		**ER-β**	**-**	**↓, *P* = 0.010**	**↑, *P* = 0.001**	**-**	**↑, *P* = 0.007**
**HIPP** **Declarative memory and neurogenesis**	**DG**	**AR**	**↓, *P* = 0.001**	**↑, *P* = 0.001**	**↑, *P* = 0.001**	**-**	**-**
		**PR**	**-**	**-**	**↑, *P* = 0.001**	**↑, *P* = 0.001**	**-**
		**ER-β**	**-**	**-**	**-**	**-**	**-**
	**CA3**	**AR**	**↓, *P* = 0.001**	**↑, *P* = 0.001**	**↑, *P* = 0.001**	**-**	**-**
		**PR**	**↓, *P* = 0.001**	**↑, *P* = 0.001**	**↓, *P* = 0.010**	**↑, *P* = 0.001**	**-**
		**ER-β**	**↑, *P* = 0.003**	**-**	**-**	**-**	**-**
	**CA1**	**AR**	**↓, *P* = 0.001**	**↓, *P* = 0.001**	**↑, *P* = 0.001**	**↑, *P* = 0.002**	**-**
		**PR**	**↓, *P* = 0.003**	**↓, *P* = 0.001**	**↑, *P* = 0.001**	**↑, *P* = 0.001**	**-**
		**ER-β**	**-**	**-**	**-**	**-**	**-**

*(A) effect of ovariectomy, (B) effect of chronic stress on NON-OVX animals, (C) effect of chronic stress on OVX animals, (D) difference between chronic stress response in NON-OVX and OVX, (E) cumulative effect of ovariectomy and chronic stress response, ↑ = significant increase; ↓ = significant decrease. Statistically significant differences were determined by Mann-Whitney test with statistical significance level *P* < 0.01. Abbreviations: AR – androgen receptor, ARC – arcuate nucleus of hypothalamus, C – control group, CA1 – Cornu Ammonis region 1, CA3 – Cornu Ammonis region 3, CTX – cortex, DG – dentate gyrus, ER-β – estrogen receptor beta, HIPP – hippocampus, HTH – hypothalamus, LH – lateral nucleus of hypothalamus, NON -OVX – non-ovariectomized animals, OVX – ovariectomized animals, PIR – piriform cortex, PR – progesterone receptor, PV – paraventricular nucleus of hypothalamus, S – chronic stress group, SNC – substantia nigra pars compacta, VTA – ventral tegmental area.

**Table 2:** The evaluation of change in expression level of leptin receptor

		NON-OVX-C vs OVX-C, (A)*	NON-OVX-C vs NON OVX-S, (B)*	OVX-C vs OVX-S, (C)*	NON-OVX-S vs OVX-S, (D)*	NON-OVX-C vs OVX-S, (E)*
**HTH**	**ARC**	**↓, *P* = 0.004**	**-**	**↑, *P* = 0.001**	**-**	**↑, *P* = 0.003**
	**LH**	**-**	**↓, *P* = 0.001**	**-**	**↑, *P* = 0.003**	**-**
	**PV**	**-**	**↓, *P* = 0.003**	**↓, *P* = 0.004**	**-**	**↑, *P* = 0.006**
**VTA**		**↑, *P* = 0.002**	**↓, *P* = 0.001**	**-**	**-**	**↓, *P* = 0.010**
**CTX**	**PIR**	**-**	**-**	**-**	**-**	**-**
**SNC**		**-**	**-**	**-**	**-**	**-**
**HIPP**	**DG**	**-**	**-**	**-**	**-**	**-**
	**CA3**	**↑, *P* = 0.005**	**-**	**-**	**-**	**-**
	**CA1**	**-**	**-**	**↑, *P* = 0.003**	**↑, *P* = 0.003**	**-**

*(A) effect of ovariectomy, (B) effect of chronic stress on NON-OVX animals, (C) effect of chronic stress on OVX animals, (D) difference between chronic stress response in NON-OVX and OVX, (E) cumulative effect of ovariectomy and chronic stress response, ↑ = significant increase; ↓ = significant decrease. Statistically significant differences were determined by Mann-Whitney test with statistical significance level *P* < 0.01. Abbreviations: ARC – arcuate nucleus of hypothalamus, C – control group, CA1 – Cornu Ammonis region 1, CA3 – Cornu Ammonis region 3, CTX – cortex, DG – dentate gyrus, HIPP – hippocampus, HTH – hypothalamus, LH – lateral nucleus of hypothalamus, NON -OVX – non-ovariectomized animals, OVX – ovariectomized animals, PIR – piriform cortex, PV – paraventricular nucleus of hypothalamus, S – chronic stress group, SNC – substantia nigra pars compacta, VTA – ventral tegmental area.

**Table 3:** The evaluation of change in expression level of insulin receptor α

		NON-OVX-C vs OVX-C, (A)*	NON-OVX-C vs NON OVX-S, (B)*	OVX-C vs OVX-S, (C)*	NON-OVX-S vs OVX-S, (D)*	NON-OVX-C vs OVX-S, (E)*
**HTH**	**ARC**	**-**	**↑, *P* = 0.001**	**↓, *P* = 0.010**	**↓, *P* = 0.001**	**-**
	**LH**	**↓, *P* = 0.001**	**-**	**-**	**-**	**↓, *P* = 0.004**
	**PV**	**-**	**-**	**-**	**-**	**-**
**VTA**		**-**	**-**	**-**	**-**	**-**
**CTX**	**PIR**	**↑, *P* = 0.008**	**↑, *P* = 0.001**	**-**	**↓, *P* = 0.001**	**-**
**SNC**		**-**	**-**	**-**	**-**	**↑, *P* = 0.002**
**HIPP**	**DG**	**↑, *P* = 0.001**	**↑, *P* = 0.001**	**↑, *P* = 0.001**	**-**	**↑, *P* = 0.002**
	**CA3**	**↓, *P* = 0.001**	**↑, *P* = 0.001**	**-**	**↓, *P* = 0.001**	**↑, *P* = 0.005**
	**CA1**	**↑, *P* = 0.010**	**↑, *P* = 0.001**	**↑, *P* = 0.001**	**↑, *P* = 0.001**	**↑, *P* = 0.001**

*(A) effect of ovariectomy, (B) effect of chronic stress on NON-OVX animals, (C) effect of chronic stress on OVX animals, (D) difference between chronic stress response in NON-OVX and OVX, (E) cumulative effect of ovariectomy and chronic stress response, ↑ = significant increase; ↓ = significant decrease. Statistically significant differences were determined by Mann-Whitney test with statistical significance level *P* < 0.01. Abbreviations: ARC – arcuate nucleus of hypothalamus, C – control group, CA1 – Cornu Ammonis region 1, CA3 – Cornu Ammonis region 3, CTX – cortex, DG – dentate gyrus, HIPP – hippocampus, HTH – hypothalamus, LH – lateral nucleus of hypothalamus, NON -OVX – non-ovariectomized animals, OVX – ovariectomized animals, PIR – piriform cortex, PV – paraventricular nucleus of hypothalamus, S – chronic stress group, SNC – substantia nigra pars compacta, VTA – ventral tegmental area.

### Distribution of analyzed receptors in HTH regions in charge of energy stores for the sake of homeostasis maintenance

In the HTH, three sub-regions were analyzed: arcuate nucleus (ARC), lateral hypothalamic nucleus (LH), and paraventricular nucleus (PV). ARC interprets leptin and insulin signaling to PV and LH. PV further activates either rise in energy expenditure or decrease in food intake. Contrary, LH activates programs that encourage eating (11).

**Figure 2:**
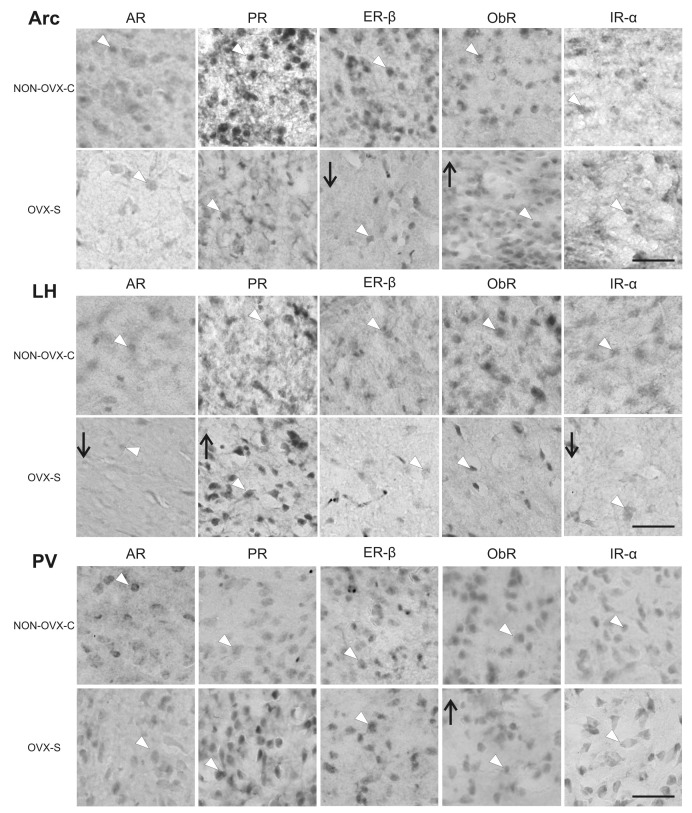
Combination of ovariectomy and chronic stress causes changes in AR, PR, ER-β, ObR, and IR-α expression level in HTH. The arrows indicate the direction of the change if significant. White triangles indicate immunopositive cells. Magnification 200 × ; scale 50 µm; area 0.02 mm^2^. Abbreviations: HTH – hypothalamus; Arc – Arcuate nucleus of hypothalamus; LH – lateral hypothalamic nucleus, PV – paraventricular nucleus of hypothalamus; NON-OVX – non-ovariectomized animals; OVX – ovariectomized animals; C – control; S – chronic stress; AR - androgen receptor; PR – progesterone receptor; ER-β – estrogen receptor beta; ObR- leptin receptor; IR-α –insulin receptor alpha.

*Cumulative effect of ovariectomy and stress was upregulation of PR in LH and downregulation of ER-β in ARC.* (A) The general effect of ovariectomy was downregulation of AR in PV and LH, and upregulation of ER-β in PV. (B) Chronic stress caused upregulation of AR in ARC and PV (Supplemental Table 1)(web extra material 1). (C) Ovariectomized female rats under the stress also increased AR expression only in PV. Upregulation of PR in LH was typical for ovariectomized females and became significant after chronic stress. (D) Two chronically stressed groups of animals were significantly different in ability to raise AR levels in satiety centers upon chronic stress; ovariectomized females successfully raised AR just in PV (Table 1). (E) When combined, chronic stress and ovariectomy downregulated ER-β in ARC, and AR in LH (Figure 2). Oppositely, upregulation of PR (in LH) after ovariectomy became even higher after additional impact of chronic stress (Supplemental Table 2)(web extra material 2).

As a general rule, ovariectomy downregulated AR in hypothalamic satiety centers, while chronic stress upregulated same receptor. Ovariectomy dictated direction of gonadal receptor expression under condition of chronic stress, which was particularly clear in rise of PR in LH. Effect of chronic stress overrode ovariectomy just in the case of upregulation of AR in PV. Ovariectomy and chronic stress worked in conjunction to downregulate ER-β in ARC.

*Ovariectomy or stress downregulated ObR in satiety centers, while combination of these two led toward paradoxical up-regulation.* (A) Ovariectomy alone downregulated ObR in ARC, while (B) just exposure to chronic stress downregulated the expression level of ObR in LH and PV. Paradoxically, the combination of stress and ovariectomy (E) caused ObR upregulation within ARC and PV, which might be a sign of developing leptin resistance (Figure 2). Since ovariectomy produced a change in the expression level of ObR just upon chronic stress (C), it is implied that ovariectomy was the key factor in ObR upregulation within ARC and PV upon chronic stress (Table 2).

(A) Ovariectomy affected IR-α in LH (downregulation), while (B) chronic stress affected more IR-α in ARC (up-regulation). In both nuclei, combined effect of stress and ovariectomy was downregulation of IR-α, more influenced by ovariectomy than stress (Table 3).

General effect of chronic stress on satiety centers was downregulation of ObR, which could be an explanation for decreased food intake in stressed animals. Although ovariectomy also induced similar changes, combination of ovariectomy and stress paradoxically caused an increase in ObR, particularly in ARC – the major satiety center and PV, a nucleus that determines the overall food intake. This change is a probable sign of leptin resistance, which triggers further deregulation.

### Distribution of analyzed receptors in CTX region involved in impression about food and VTA region included in feeding for reward pathway

One sub-region of CTX was analyzed – the piriform cortex (PIR), which is involved in perception of smell. It may have an important role in the motivation of animals to eat even if the animal does not have the need for extra energy intake. The same region suffers the first neurodegenerative changes and is probably the most sensitive to changes under conditions of ovariectomy or chronic stress.

VTA represent dopaminergic areas which may be implicated in control of reward pathway based on food and food related stimuli. The signals from this region can override the control of energy stores and motivate the animal to eat and thus lead to extra energy intake.

**Figure 3:**
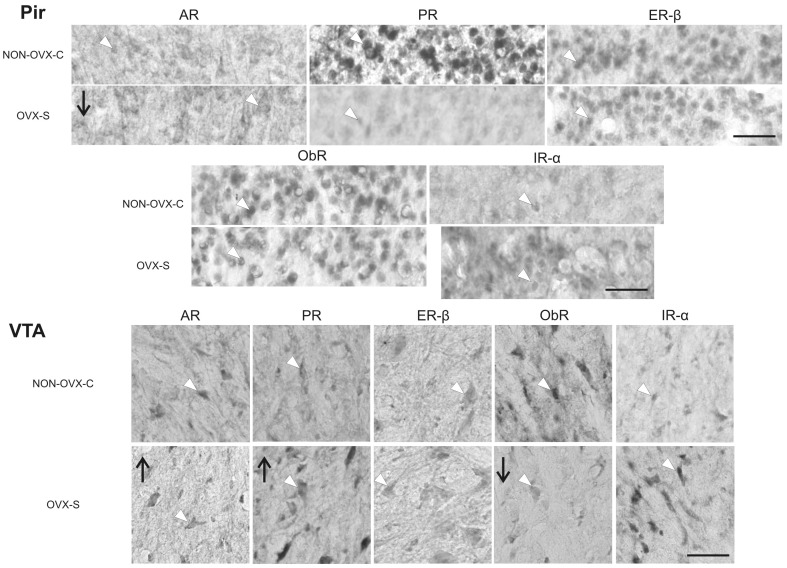
Combination of ovariectomy and chronic stress causes changes in AR, PR, ER-β, ObR, and IR-α expression level in PIR and VTA. The arrows indicate direction of the change if significant. Triangles indicate immunopositive cells. Magnification 200x; scale 50 µm; area 0.02 mm^2^. Abbreviations: PIR – piriform cortex; VTA – ventral tegmental area; NON-OVX – non-ovariectomized animals; OVX – ovariectomized animals; C – control; S – chronic stress; AR – androgen receptor; PR – progesterone receptor; ER-β – estrogen receptor beta; ObR- leptin receptor; IR-α –insulin receptor alpha.

*Combination of ovariectomy and stress upregulated expression of AR and PR in VTA.* (A) Ovariectomy caused downregulation of AR in PIR. Contrary to hypothalamic satiety centers and PIR, ovariectomy upregulated AR and PR in VTA. This was the only region in which AR increased right after ovariectomy (Table 1). Also, PR increased immediately after ovariectomy in this region, but it increased even more after additional chronic stress (C) (Supplemental Table 2)(web extra material 2). Upregulation of PR due to stress in ovariectomized females was previously observed in LH and commented as specific for them. (C) Stress response of ovariectomized females was downregulation of AR and upregulation of PR and ER-β in PIR. However, ER-β was upregulated to the levels observed in NON-OVX-C animals (Supplemental Table 3)(web extra material 3). (D) Ovariectomized females upon chronic stress exposure ended up with much higher PR levels in VTA than non-ovariectomized ones (Table 1). (E) Combination of ovariectomy and chronic stress in VTA upregulated PR, which was already observed in LH (Figure 3). Also, combination of ovariectomy and stress in PIR downregulated the expression of AR, like in LH.

VTA had different response to ovariectomy and chronic stress than HTH satiety regions – it maintained AR and upregulated PR levels at the same time. Different response might generate counterbalance to satiety regions in affective food perception and lead toward higher gratification to food.

*Combined effect of stress and ovariectomy in VTA was downregulation of ObR. *(A) ObR levels increased in VTA upon ovariectomy, but (B) decreased upon chronic stress (Table 2). (E) Combined effect of ovariectomy and chronic stress was down-regulation of ObR – contrary to the final effect in HTH satiety centers.

### Distribution of analyzed receptors in regions for declarative (HIPP) and non-declarative (SNC) memory

In the HIPP, three sub-regions were analyzed: dentate gyrus (DG), and two *Cornu Ammonis* regions – CA1 and CA3. These regions are involved in learning and declarative memory management. Also, neurogenesis is proved to occur in DG. SNC is a dopaminergic region involved in non-declarative memory. We were interested if dysregulation of energy expenditure under conditions of ovariectomy and/or chronic stress could explain the susceptibility of memory regions toward neurodegeneration.

**Figure 4:**
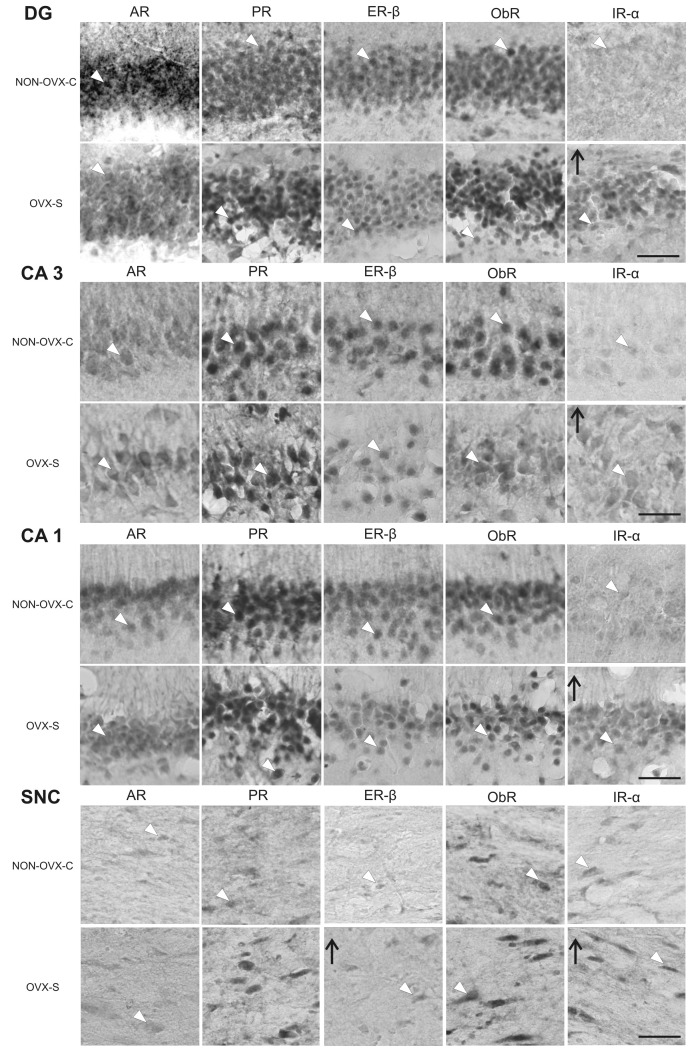
Combination of ovariectomy and chronic stress causes changes in AR, PR, ER-β ObR and IR-α expression level in non-declarative (SCN) and declarative (HIPP) memory regions. The arrows indicate the direction of the change if significant. Triangles indicate immunopositive cells. Magnification 200x; scale 50 µm; area 0.02 mm^2^. Abbreviations: SCN – *substantia nigra pars compacta*; HIPP-Hippocampus; DG – dentate gyrus; CA1 – *Cornu Ammonis* region 1; CA3 – *Cornu Ammonis* region 3; NON-OVX – non-ovariectomized animals; OVX – ovariectomized animals; C – control; S – chronic stress; AR - androgen receptor; PR – progesterone receptor; ER-β – estrogen receptor beta; ObR- leptin receptor; IR-α –insulin receptor alpha.

*Ovariectomy and stress had opposite effects on steroid gonadal receptors expression in HIPP and in combination they canceled the effects of each other.* (A) General effect of ovariectomy was downregulation of AR in all HIPP sub-regions, and PR in CA regions. Ovariectomy caused rise of ER-β in CA3. (B) Chronic stress had same effect as ovariectomy on CA1, but opposite effect in DG and CA3. At the same time (E), combination of stress and ovariectomy canceled the influence of each other on gonadal steroid receptors expression in all HIPP sub-regions (Figure 4).

(B) Chronic stress upregulated PR expression in SNC, which was also observed in CA3 and was a typical effect of stress after ovariectomy in HTH. (C) Ovariectomy inverted stress response within SNC and (E) combination of ovariectomy and chronic stress brought the levels of steroid gonadal receptors to the control values (Supplemental Tables 1, 2 and 3). We can conclude that stress and ovariectomy within SNC acted oppositely and they mostly annulled each other’s influence.

*Ovariectomy and chronic stress lead toward significant upregulation of IR-α in all HIPP regions and SNC, but had no effect on ObR in HIPP.* (A) ObR was upregulated in CA3 due to ovariectomy, while (B) individually chronic stress had no impact on its expression in HIPP or SNC (Table 2). In ovariectomized females chronic stress upregulated ObR in CA1 (C). Finally, the levels of ObR in HIPP and SNC were not affected upon combined ovariectomy and stress (Supplemental Table 4)(web extra material 4).

Ovariectomy (A) and chronic stress individually (B), and in combination (E) caused significant IR-α upregulation in HIPP sub-regions and SNC (Table 3 and Supplemental Table 5)(web extra material 5). These results imply HIPP and SNC sensitivity to development of insulin resistance in case of ovariectomy and chronic stress.

## Discussion

Results of this study showed that ovariectomy and chronic stress affected the expression of gonadal steroid, leptin, and insulin receptors in the rat brain. These effects were analyzed in the hypothalamic regions involved in control of satiety and dopaminergic areas involved in control of feeding for reward and non-declarative memory. Furthermore, they were analyzed in the cortical region, involved in impression about food and feeding motivation (25), and the hippocampus, a brain structure that manages declarative learning and memory (26,27) and provides environment for neurogenesis (28).

### Ovariectomy downregulated gonadal steroid receptors and prevented or attenuated stress specific upregulation of AR

Ovariectomy caused downregulation of AR in hypothalamic regions that mediate promoting or inhibiting the signal for energy intake. Studies have shown that AR is related to anxiety behaviors in rats. Increased AR activation inhibits stress response and *vice versa*. Knockout mice that lack the androgen receptors show increased HPA activation (29,30). However, there are no similar data on testosterone and progesterone receptors after ovariectomy. Our conclusion is that not just downregulation of AR but also the rise of PR might serve as a marker of ovariectomy and be the underlying cause of physiological changes of satiety regions, particularly under conditions of chronic stress.

The effect of chronic stress on animals in reproductive age in our study was estimated by comparing NON-OVX-S with NON-OVX-C group. Stress caused an increase in AR in ARC and PV. It seems that physiology of ARC after ovariectomy is characterized by inability to increase AR, particularly in chronic stress response. Since ARC is the satiety-regulating brain center we concluded that this combined effect reflected on feeding behavior and body weight. In our previous study non-ovariectomized animals exposed to chronic stress kept constant weight during the stress period (23). It was unexpected, because some previous studies reported weight loss under the chronic stress (31). We suppose that the rise of AR in ARC during reproductive age of females is a protective factor under conditions of stress which helps in maintaining constant weight. At the same time animals that were ovariectomized gained body weight in spite of stress.

Ovariectomy downregulated AR in PIR region. We still have to explore if change in expression of AR affect animal behavior in direction of looking for different source of food. In general, there are no studies exploring animal’s affinity toward certain taste of food under conditions of chronic stress.

Ovariectomy downregulated AR and PR in all regions of HIPP, with exception of PR in DG. On the other hand chronic stress in non-ovariectomized animals caused increase in all gonadal steroid hormones in DG and CA3. Chronic stress had such an impact on DG and CA3 region that even ovariectomized animals after chronic stress successfully upregulated all gonadal steroid receptors, except PR in CA3. Interestingly, CA1 sub-region differs in response to chronic stress; instead of rise of AR and PR we observed downregulation, like in ovariectomy. Even more interesting is that individual effects of ovariectomy and chronic stress (overall downregulation) in CA1 became completely inverted if combined and we saw overall upregulation of gonadal steroid receptors after chronic stress even in this region. Most studies dealing with the effect of reproductive hormones on hippocampal tissue overlook a possible role of PR. Our observation of significant changes induced in expression level of PR after ovariectomy and stress indicate a possible role of progesterone in the regulation of stress response in hippocampus.

### Ovariectomy-induced and chronic stress-induced effects on the expression of leptin and insulin receptors

Ovariectomy downregulated the levels of ObR in ARC, while chronic stress downregulated ObR in LH and PV. On the other hand, if we exposed ovariectomized animals to additional stress, levels of ObR ended up significantly upregulated in ARC and PV. Mesencephalic gratification region VTA reacted in the opposite way, upregulated ObR after ovariectomy, downregulated upon chronic stress and in case of both ended up with downregulation. Satiety regions are more likely to respond to a variety of stressors with changes in ObR levels than any other brain region. Due to the fact that the hypothalamus regulates autonomic nervous functions and controls overall body energy balance (32,33), we might expect profound long-term changes in the regulation of body weight in combination of stress and ovariectomy.

Ovariectomy alone, but also chronic stress alone, significantly elevates the level of IR-α in all analyzed regions known for being susceptible to early neurodegeneration (PIR, SNC, HIPP). Satiety regions are spared from fluctuation in IR-α and are probably not susceptible to insulin resistance. We can imply the possibility that rise in IR-α alone could be a good predictor of insulin resistance, but further functional studies are required for clarification. In the light of the recently discovered connection between neurodegeneration and insulin resistance (34), our results might point to the chronic stress exacerbated by gonadal hormone deprivation as a possible cause of diabetes-C (35).

The lack of ovariectomized animal group with estrogen replacement therapy (ERT) might be considered as limitation of this study. Considering the fact that exogenous estrogen also influences HPA axis, we did not include this animal group in the study because ERT would introduce additional stress (36), making this group incomparable with other groups. Besides, it has been shown that after ovariectomy the levels of endogenous estrogen are slowly being restored by other peripheral tissues (37). 

In conclusion, our data suggested that ovariectomy in general downregulated the levels of gonadal steroid receptors with exception of VTA. The general effect of chronic stress response was rise of AR and PR in the brain of female rats in the period of reproductive life. When combined with ovariectomy, stress response nullified ovariectomy and brought levels of steroid hormone receptors to those common for age or even higher.

While ovariectomy downregulated the levels of ObR in ARC, chronic stress brought down ObR in PV and LH. Combination of ovariectomy and stress reversed individual effects and led toward significant upregulation of ObR in hypothalamic satiety centers, but not in VTA, which probably works as counterbalance. The most significant finding of our study is the possible link between chronic stress response (amplified by ovariectomy) and development of insulin resistance in the hippocampus and other brain regions susceptible to early neurodegeneration.

Described effects of chronic stress and gonadal steroid hormone deprivation were assessed in adult female Sprague-Dawley rats in the middle of reproductive age (38). Since AR notably changed in females, it would be interesting to clarify whether the observed differences under the same conditions could be observed in male counterparts or not. Also, further studies might reveal the alteration of chronic stress response of aged females in the reproductive senescence period. Finally, to determine the implications of observed differences, functional studies on cell lines with overexpressed IR and/or ObR are needed.
