# Supplementary material for: Ovariectomy and chronic stress lead toward leptin resistance in the satiety centers and insulin resistance in the hippocampus of Sprague-Dawley rats
Source: Croat Med J. 2016 Apr;57(2):194–206. doi: 10.3325/cmj.2016.57.194 (PMC4856194; doi:10.3325/cmj.2016.57.194)
Supplement: Supplementary Table 1 [file CroatMedJ_57_s001.pdf]

Supplementary Table 1. Median with IQR for AR in selected brain regions.

AR						
		MINIMUM	Q_1	MEDIAN	Q_3	MAXIMUM
animal group	**NON-OVX-C**					
brain region	ARC	12.00	15.75	20.50	24.00	28.00
	LH	15.00	15.75	20.00	21.50	24.00
	PV	17.00	21.75	23.00	25.00	26.00
	VTA	7.00	9.00	10.00	12.50	15.00
	PIR	36.00	43.00	45.00	71.00	74.00
	SNC	9.00	10.75	13.00	14.00	17.00
	DG	49.00	50.00	51.00	51.50	52.00
	CA3	17.00	18.00	19.00	19.50	20.00
	CA1	27.00	28.00	29.00	30.50	32.00
animal group	**OVX-C**					
brain region	ARC	13.00	16.00	21.00	23.00	27.00
	LH	8.00	10.00	12.00	14.00	20.00
	PV	15.00	16.50	18.50	21.00	24.00
	VTA	11.00	14.75	16.00	17.25	18.00
	PIR	16.00	18.00	26.00	32.25	36.00
	SNC	11.00	12.75	16.00	17.25	19.00
	DG	7.00	7.50	8.00	10.00	12.00
	CA3	3.00	7.50	12.00	12.50	13.00
	CA1	5.00	5.50	6.00	7.00	8.00
animal group	**NON-OVX-S**					
brain region	ARC	33.00	36.25	37.00	38.50	46.00
	LH	10.00	12.50	17.00	21.25	24.00
	PV	13.00	15.75	17.00	20.00	24.00
	VTA	10.00	12.00	14.00	16.00	17.00
	PIR	19.00	26.00	27.00	41.00	47.00
	SNC	10.00	10.00	12.00	13.00	15.00
	DG	53.00	53.50	54.00	54.00	54.00
	CA3	19.00	19.50	20.00	22.00	24.00
	CA1	21.00	21.00	21.00	21.50	22.00
animal group	**OVX-S**					
brain region	ARC	15.00	16.00	16.00	16.00	19.00
	LH	6.00	10.00	11.00	15.00	16.00
	PV	20.00	23.00	25.00	28.00	29.00
	VTA	14.00	14.50	17.00	19.50	20.00
	PIR	30.00	32.00	35.00	37.00	41.00

	SNC	9.00	11.00	13.50	15.00	18.00
	DG	17.00	18.00	19.00	20.00	21.00
	CA3	17.00	17.50	18.00	20.00	22.00
	CA1	31.00	32.50	34.00	34.00	34.00

Abbreviations: AR – androgen receptor, ARC – arcuate nucleus of hypothalamus, C – control group, CA1 – *Cornu Ammonis* region 1, CA3 – *Cornu Ammonis* region 3, DG – dentate gyrus, IQR – interquartile range, LH – lateral nucleus of hypothalamus, NON-OVX – non-ovariectomized animals, OVX – ovariectomized animals, PIR – piriform cortex, PV – paraventricular nucleus of hypothalamus, Q1 – first quartile, Q3 – third quartile, S – chronic stress group, SNC – *substantia nigra pars compacta,* VTA – ventral tegmental area.
