# Supplementary material for: Ovariectomy and chronic stress lead toward leptin resistance in the satiety centers and insulin resistance in the hippocampus of Sprague-Dawley rats
Source: Croat Med J. 2016 Apr;57(2):194–206. doi: 10.3325/cmj.2016.57.194 (PMC4856194; doi:10.3325/cmj.2016.57.194)
Supplement: Supplementary Table 2 [file CroatMedJ_57_s002.pdf]

Supplementary Table 2. Median with IQR for PR in selected brain regions.

PR						
		MINIMUM	Q_1	MEDIAN	Q_3	MAXIMUM
animal group	NON-OVX-C					
brain region	ARC	35.00	37.00	39.00	41.00	48.00
	LH	10.00	16.50	19.50	23.50	29.00
	PV	21.00	23.50	25.50	31.25	37.00
	VTA	16.00	16.00	20.00	20.00	24.00
	PIR	36.00	38.00	57.00	74.00	85.00
	SNC	18.00	21.00	23.00	25.00	27.00
	DG	43.00	43.50	44.00	44.50	45.00
	CA3	18.00	18.50	19.00	19.00	19.00
	CA1	26.00	26.50	27.00	27.50	28.00
animal group	OVX-C					
brain region	ARC	32.00	34.00	36.00	40.00	49.00
	LH	20.00	20.00	23.50	26.00	30.00
	PV	30.00	30.00	31.50	34.50	44.00
	VTA	18.00	21.00	23.00	25.00	25.00
	PIR	32.00	33.00	39.50	55.75	75.00
	SNC	17.00	19.00	19.00	23.00	27.00
	DG	40.00	42.00	44.00	44.00	44.00
	CA3	19.00	20.00	21.00	21.50	22.00
	CA1	12.00	12.00	12.00	13.00	14.00
animal group	NON-OVX-S					
brain region	ARC	13.00	14.25	30.50	46.00	48.00
	LH	11.00	13.25	16.00	18.75	23.00
	PV	26.00	27.75	28.00	28.25	30.00
	VTA	18.00	20.00	23.00	25.00	27.00
	PIR	21.00	31.25	44.00	53.00	58.00
	SNC	15.00	18.00	18.00	20.00	20.00
	DG	34.00	34.00	34.00	37.50	41.00
	CA3	19.00	19.50	20.00	20.50	21.00
	CA1	16.00	16.50	17.00	18.50	20.00
animal group	OVX-S					
brain region	ARC	24.00	29.00	31.00	44.00	54.00
	LH	25.00	30.00	32.00	41.00	46.00
	PV	27.00	28.00	32.00	33.00	35.00
	VTA	24.00	25.00	27.00	29.00	33.00
	PIR	28.00	37.25	46.00	54.75	64.00
	SNC	18.00	19.00	21.00	22.00	23.00
	DG	43.00	46.50	50.00	57.00	64.00

	CA3	18.00	18.50	19.00	19.50	20.00
	CA1	28.00	29.00	30.00	30.50	31.00

Abbreviations: ARC – arcuate nucleus of hypothalamus, C – control group, CA1 – *Cornu Ammonis* region 1, CA3 – *Cornu Ammonis* region 3, DG – dentate gyrus, IQR – interquartile range, LH – lateral nucleus of hypothalamus, NON-OVX – non-ovariectomized animals, OVX – ovariectomized animals, PIR – piriform cortex, PR – progesterone receptor, PV – paraventricular nucleus of hypothalamus, Q1 – first quartile, Q3 – third quartile, S – chronic stress group, SNC – *substantia nigra pars compacta,* VTA – ventral tegmental area.
