# Supplementary material for: Ovariectomy and chronic stress lead toward leptin resistance in the satiety centers and insulin resistance in the hippocampus of Sprague-Dawley rats
Source: Croat Med J. 2016 Apr;57(2):194–206. doi: 10.3325/cmj.2016.57.194 (PMC4856194; doi:10.3325/cmj.2016.57.194)
Supplement: Supplementary Table 3 [file CroatMedJ_57_s003.pdf]

Supplementary Table 3. Median with IQR for ER-β in selected brain regions.

ER-β						
		MINIMUM	Q_1	MEDIAN	Q_3	MAXIMUM
animal group	NON-OVX-C					
brain region	ARC	28.00	30.00	48.00	51.00	55.00
	LH	12.00	14.75	22.50	26.00	33.00
	PV	27.00	33.00	35.00	39.00	41.00
	VTA	30.00	33.00	34.00	36.50	38.00
	PIR	23.00	29.00	39.50	57.25	68.00
	SNC	31.00	32.00	33.00	37.00	39.00
	DG	20.00	20.50	29.00	36.75	38.00
	CA3	11.00	12.50	14.00	14.00	14.00
	CA1	10.00	12.00	17.00	22.00	24.00
animal group	OVX-C					
brain region	ARC	14.00	19.50	38.50	59.00	60.00
	LH	11.00	15.75	17.00	19.25	22.00
	PV	20.00	21.75	23.00	29.25	31.00
	VTA	14.00	19.00	21.00	24.00	28.00
	PIR	17.00	22.75	42.50	60.25	67.00
	SNC	30.00	35.00	37.00	38.00	41.00
	DG	42.00	44.25	45.50	46.75	48.00
	CA3	15.00	15.25	18.00	21.50	23.00
	CA1	10.00	11.25	14.50	17.75	21.00
animal group	NON-OVX-S					
brain region	ARC	27.00	35.00	36.00	38.00	46.00
	LH	8.00	10.00	11.00	18.00	23.00
	PV	25.00	32.00	34.50	36.00	41.00
	VTA	30.00	33.00	36.00	36.00	38.00
	PIR	26.00	38.25	45.00	52.25	66.00
	SNC	26.00	28.00	28.00	31.00	35.00
	DG	30.00	38.25	45.00	48.75	52.00
	CA3	14.00	14.50	17.00	19.50	22.00
	CA1	16.00	16.00	16.00	16.75	18.00
animal group	OVX-S					
brain region	ARC	21.00	26.00	27.00	28.00	31.00
	LH	13.00	14.00	15.50	19.00	24.00
	PV	25.00	28.00	30.50	35.25	38.00
	VTA	28.00	29.00	33.00	36.00	38.00
	PIR	28.00	35.75	42.00	51.00	74.00
	SNC	22.00	25.00	30.00	32.00	33.00
	DG	43.00	45.00	46.00	47.00	49.00

	CA3	7.00	9.00	12.50	16.75	18.00
	CA1	13.00	14.00	19.00	24.00	24.00

Abbreviations: ARC – arcuate nucleus of hypothalamus, C – control group, CA1 – *Cornu Ammonis* region 1, CA3 – *Cornu Ammonis* region 3, DG – dentate gyrus, ER-β – estrogen receptor beta, IQR – interquartile range, LH – lateral nucleus of hypothalamus, NON-OVX – non-ovariectomized animals, OVX – ovariectomized animals, PIR – piriform cortex, PV – paraventricular nucleus of hypothalamus, Q1 – first quartile, Q3 – third quartile, S – chronic stress group, SNC – *substantia nigra pars compacta,* VTA – ventral tegmental area.
