# Supplementary material for: Ovariectomy and chronic stress lead toward leptin resistance in the satiety centers and insulin resistance in the hippocampus of Sprague-Dawley rats
Source: Croat Med J. 2016 Apr;57(2):194–206. doi: 10.3325/cmj.2016.57.194 (PMC4856194; doi:10.3325/cmj.2016.57.194)
Supplement: Supplementary Table 4 [file CroatMedJ_57_s004.pdf]

Supplementary Table 4. Median with IQR for ObR in selected brain regions.

ObR						
		MINIMUM	Q_1	MEDIAN	Q_3	MAXIMUM
animal group	**NON-OVX-C**					
brain region	ARC	47.00	48.25	49.00	52.00	53.00
	LH	13.00	16.00	17.00	18.00	19.00
	PV	27.00	31.75	32.00	35.25	41.00
	VTA	7.00	9.00	12.00	13.00	15.00
	PIR	39.00	43.00	50.00	51.00	52.00
	SNC	10.00	12.00	12.00	15.00	18.00
	DG	12.00	18.00	24.00	30.00	34.00
	CA3	8.00	8.00	10.00	13.50	14.00
	CA1	9.00	9.25	16.50	23.75	26.00
animal group	**OVX-C**					
brain region	ARC	43.00	43.25	44.00	44.75	45.00
	LH	14.00	15.00	15.00	18.00	19.00
	PV	26.00	28.50	32.00	34.00	42.00
	VTA	11.00	15.00	16.00	19.00	24.00
	PIR	45.00	50.00	50.00	51.00	54.00
	SNC	10.00	15.00	17.00	18.00	19.00
	DG	20.00	21.25	28.00	36.25	44.00
	CA3	14.00	15.25	16.00	16.00	16.00
	CA1	14.00	14.00	14.00	14.75	15.00
animal group	**NON-OVX-S**					
brain region	ARC	31.00	34.75	40.50	46.25	53.00
	LH	8.00	8.25	9.00	9.00	9.00
	PV	31.00	39.00	42.00	45.25	47.00
	VTA	15.00	16.00	16.00	18.00	28.00
	PIR	48.00	52.25	53.00	53.75	58.00
	SNC	13.00	14.00	15.00	17.00	20.00
	DG	23.00	25.50	36.00	45.75	47.00
	CA3	12.00	12.00	12.50	13.75	14.00
	CA1	14.00	15.00	15.00	15.00	15.00
animal group	**OVX-S**					
brain region	ARC	50.00	56.50	64.50	71.00	78.00
	LH	12.00	13.50	16.00	17.75	18.00
	PV	35.00	35.00	40.00	42.00	45.00
	VTA	12.00	13.00	18.00	20.00	27.00
	PIR	45.00	46.50	49.00	52.25	53.00
	SNC	11.00	14.00	14.00	15.00	18.00
	DG	34.00	36.25	40.00	44.50	47.00

	CA3	13.00	13.25	15.00	17.50	18.00
	CA1	22.00	22.00	23.00	24.75	26.00

Abbreviations: ARC – arcuate nucleus of hypothalamus, C – control group, CA1 – *Cornu Ammonis* region 1, CA3 – *Cornu Ammonis* region 3, DG – dentate gyrus, IQR – interquartile range, LH – lateral nucleus of hypothalamus, NON-OVX – non-ovariectomized animals, ObR – leptin receptor, OVX – ovariectomized animals, PIR – piriform cortex, PV – paraventricular nucleus of hypothalamus, Q1 – first quartile, Q3 – third quartile, S – chronic stress group, SNC – *substantia nigra pars compacta,* VTA – ventral tegmental area.
