# Supplementary material for: Ovariectomy and chronic stress lead toward leptin resistance in the satiety centers and insulin resistance in the hippocampus of Sprague-Dawley rats
Source: Croat Med J. 2016 Apr;57(2):194–206. doi: 10.3325/cmj.2016.57.194 (PMC4856194; doi:10.3325/cmj.2016.57.194)
Supplement: Supplementary Table 5 [file CroatMedJ_57_s005.pdf]

Table 5. Median with IQR for IR-α in selected brain regions.

IR-α						
		MINIMUM	Q_1	MEDIAN	Q_3	MAXIMUM
animal group	**NON-OVX-C**					
brain region	ARC	22.00	27.00	36.00	37.00	43.00
	LH	23.00	24.00	25.00	26.00	28.00
	PV	18.00	23.00	30.00	31.00	38.00
	VTA	11.00	14.50	17.00	18.75	24.00
	PIR	37.00	42.75	48.00	50.25	54.00
	SNC	9.00	10.00	11.00	14.00	17.00
	DG	38.00	47.00	54.00	59.00	64.00
	CA3	21.00	22.00	23.00	25.00	28.00
	CA1	41.00	42.00	43.00	44.00	47.00
animal group	**OVX-C**					
brain region	ARC	27.00	32.00	34.56	38.00	41.00
	LH	14.00	18.00	19.00	21.00	24.00
	PV	25.00	28.00	29.67	31.00	37.00
	VTA	15.00	16.00	17.78	20.00	24.00
	PIR	28.00	32.00	37.78	45.00	49.00
	SNC	13.00	14.00	14.67	16.00	17.00
	DG	60.00	66.00	66.56	68.00	69.00
	CA3	27.00	28.00	30.44	32.00	35.00
	CA1	34.00	37.00	39.00	41.00	47.00
animal group	**NON-OVX-S**					
brain region	ARC	43.00	45.00	47.00	51.00	58.00
	LH	19.00	22.00	22.00	26.00	33.00
	PV	26.00	28.00	37.00	40.00	48.00
	VTA	15.00	20.00	23.00	26.00	28.00
	PIR	55.00	57.00	58.00	61.00	69.00
	SNC	11.00	13.00	14.00	20.00	23.00
	DG	61.00	66.00	71.00	77.00	87.00
	CA3	38.00	43.00	47.00	48.00	54.00
	CA1	56.00	61.00	67.00	71.00	73.00
animal group	**OVX-S**					
brain region	ARC	26.00	28.00	29.00	29.00	32.00
	LH	18.00	20.00	21.00	24.00	25.00
	PV	21.00	25.00	30.00	35.00	39.00
	VTA	17.00	17.00	19.00	22.00	24.00
	PIR	33.00	36.00	40.00	45.00	50.00
	SNC	14.00	16.00	18.00	21.00	22.00

	DG	57.00	67.00	80.00	84.00	88.00
	CA3	24.00	25.00	31.00	33.00	34.00
	CA1	46.00	48.00	49.00	51.00	53.00

Abbreviations: ARC – arcuate nucleus of hypothalamus, C – control group, CA1 – *Cornu Ammonis* region 1, CA3 – *Cornu Ammonis* region 3, DG – dentate gyrus, IQR – interquartile range, IR-α – insulin receptor alpha, LH – lateral nucleus of hypothalamus, NON-OVX – non-ovariectomized animals, OVX – ovariectomized animals, PIR – piriform cortex, PV – paraventricular nucleus of hypothalamus, Q1 – first quartile, Q3 – third quartile, S – chronic stress group, SNC – *substantia nigra pars compacta,* VTA – ventral tegmental area.
